# Highlights from Heart Rhythm Society 2017: Atrial Fibrillation

**DOI:** 10.19102/icrm.2017.080801

**Published:** 2017-08-15

**Authors:** Rahul N. Doshi


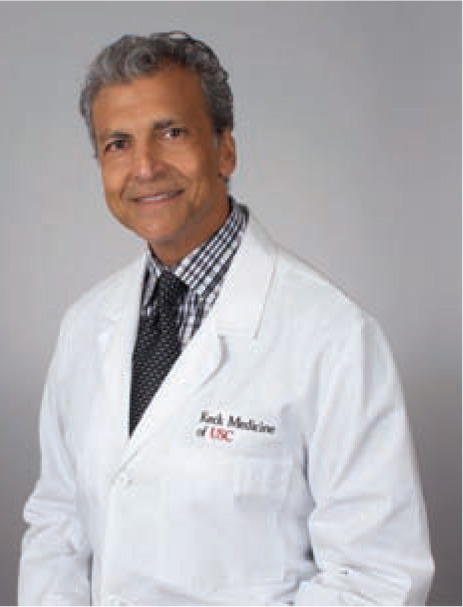


## Introduction

The Sessions Planning Committee and Chair should be congratulated for their work on the 2017 Heart Rhythm Society Annual Scientific Sessions held in Chicago, IL, as it was another phenomenal meeting. This year was an example of my favorite saying, “As so goes atrial fibrillation (AF), so goes electrophysiology”; namely, there were many fascinating aspects of AF presented during the late-breaking clinical trials, and there seems to be a shift occurring from interest in primary ablation to interest in the prevention of thromboembolism. Here are a few highlights from this year’s show.

### Left atrial appendage

The left atrial appendage (LAA) has quite possibly dethroned rotors as the hot topic in AF, both for cerebrovascular accident (CVA) prevention and in catheter ablation. SentreHEART (Palo Alto, CA, USA) announced that, regarding the Left Atrial Appendage Ligation with the LARIAT™ Suture Delivery System as Adjunctive Therapy to Pulmonary Vein Isolation for Persistent or Longstanding Persistent Atrial Fibrillation (aMAZE) trial,^1^ the study’s safety data at the time of initial review met criteria to continue the trial. There was also an elegant live case streamed from Northwestern University of combined cryoballoon (Arctic Front Advance^®^; Medtronic, Minneapolis, MN, USA) AF ablation and LARIAT™ (SentreHEART, Palo Alto, CA, USA) LAA ligation.

Dr. Lucas Boersma of St. Antonius Hospital in the Netherlands presented the results of the EWOLUTION trial^2^ on patients receiving the WATCHMAN™ device (Boston Scientific, Natick, MA, USA). The initial randomized controlled trials (RCTs) for the device involved patients who were candidates for oral anticoagulant (OAC) therapy, and the initial pivotal trials required that OAC therapy be administered for a minimum of 45 days following initial device implantation.^3,4^ The EWOLUTION trial followed more than 1,000 patients after initial implantation as part of a multicenter registry. The population in the EWOLUTION trial was a high-risk population as compared with that seen in prior RCTs with regards to both stroke and bleeding, with an average CHA2DS2-VASc score of 4.5 and with 73% of patients having some contraindication to OAC therapy. Fifty percent of patients had a CHA2DS2-VASc score of ≥ 5 and 40% had a HAS-BLED score of ≥ 4.

Consistent with the increasing experience with the device, the researchers running the trial demonstrated a lower risk of serious adverse events than what was seen in either the PROTECT or PREVAIL trials, and a high implant success rate of 98.5%. Only 27% of patients were treated with OAC post implantation, with 60% being given dual antiplatelet therapy. The researchers also demonstrated a marked reduction in CVA and bleeding compared with projected rates. They concluded that the WATCHMAN™ device can safely be used in patients who have a contraindication to OAC therapy, and demonstrated dramatic efficacy despite the majority of patients not undergoing OAC therapy post implantation. More importantly, this data paves the way for a prospective study evaluating whether or not OAC therapy can be completely avoided in patients receiving this type of therapy.

### Monitoring of AF

Dr. James Reiffel of Columbia University presented the primary results of the REVEAL-AF study,^5^ a prospective, single-arm open-label trial involving patients receiving a REVEAL^®^ (Medtronic, Minneapolis, MN, USA) implantable loop recorder who were at high risk for CVA and who had no prior history of AF. Fifty percent of the patients were implanted because of complaints of palpitations. The mean CHA2DS2-VASc score was 4.4. The primary endpoint was AF lasting more than six minutes at 18 months. The decision to use six minutes as an endpoint is based on the previously published ASSERT trial,^6^ which investigated the significance of AF detected with the use of cardiovascular implantable electronic device monitoring. In the REVEAL-AF study, 29.3% of patients were seen to have AF at 18 months, and 40% by 30 months. The authors concluded that three-quarters of patients would have gone undiagnosed if monitoring had only been continued for 30 days.

Given the tremendous morbidity of AF-associated CVA, aggressive monitoring for AF in high-risk patients is a good concept. The CRYSTAL-AF study previously demonstrated a high incidence of AF in patients with cryptogenic stroke.^7^ The REVEAL-AF study now considers this same concept applied to high-risk patients without a history of CVA. The authors also report that 56.3% of patients were started on OAC therapy based on the diagnosis of AF (non-protocol driven).

### Timing of anticoagulant therapy for AF

The group at Intermountain Health in Utah has given us further insight into the timing of OAC therapy for AF. Having previously demonstrated the relationship of AF and microemboli and clinical dementia, Dr. Jared Bunch reported data investigating the relationship of dementia risk to the timing of OAC or antiplatelet therapy once a diagnosis of AF was made. In a patient cohort of over 26,000 patients, in whom almost 22,000 were started on antiplatelet therapy and 4,400 on OAC therapy, Bunch and colleagues compared the incidence of dementia in patients who started OAC therapy within 30 days of AF diagnosis, or between 30 days and one year after AF diagnosis.^8^ Interestingly, there was a small but notable decrease in the risk of dementia when antiplatelet therapy was initiated within 30 days (0.4% vs. 0.5%, p=0.05). However, there was a profound benefit when OAC therapy with warfarin was started within 30 days of diagnosis, with no demonstrable incidence of dementia, compared with a 0.4% incidence when warfarin was started later. They demonstrate a clear temporal relationship between timing of OAC or antiplatelet therapy initiation and lifetime dementia risk.

These powerful data demonstrate the importance of not just appropriate OAC initiation but also the necessity for rapid initiation and improved patient compliance. One such method could be intermittent OAC use associated with a prompt diagnosis. The TACTIC-AF study^9^ presented during the late breaking clinical trials introduced listeners to the concept of “tailored anticoagulation,” which takes advantage of the rapid onset of non-vitamin-K oral anticoagulants (NOACs). This was a prospective, randomized controlled trial in patients with implanted CIEDs with a primary endpoint of days on OAC therapy. This trial, sponsored by Abbott Medical (Chicago, IL, USA) enrolled 61 patients with CIEDs equipped with remote monitoring and who were on stable NOAC therapy. Forty-eight of the 61 were randomized to the tailored therapy arm. Patients were monitored biweekly and were allowed to stop OAC therapy if no AF was seen for 30 days. Data collected indicated the use of NOACs decreased by 75% in the treatment arm. There was no incidence of CVA seen in the trial. Further investigation is clearly warranted, but this approach could certainly improve patient compliance and decrease cost and bleeding risk as compared with continuous therapy.

### Diagnosis with a smartwatch

The use of smartwatches by the general population is rapidly increasing and becoming as ubiquitous as smartphones (I say, as I look down at my Apple Watch). Smartphones are already being used to diagnose AF.^10^ Dr. Sanchez and his colleagues at the University of California San Francisco and Cardiogram (San Francisco, CA, USA) presented some fascinating data on the use of a smartwatch to diagnose AF. The mRhythm trial investigates the ability of a smartwatch to detect AF with use of its built-in photoplenthysmography sensors.^11^ The application goes through a “learning phase,” employing deep neural networks to predict patterns in an individual’s heart rate, and then a test phase in-person when the subjects presented for cardioversion using a Kardia smartphone ECG (AliveCor, Mountain View, CA, USA). There were approximately 6,400 subjects, of which there were 440 who had AF and were evaluated as part of a necessary phase for the system to “learn,” and 50 in the test phase who presented for cardioversion. The application demonstrated 98% sensitivity and 90% specificity in diagnosing AF. These tools that are in the realm of “body computing” can lead to treatments that are both more effective and patient-centric, and visionaries such as my partner Dr. Leslie Saxon at the University of Southern California Keck School of Medicine would predict that this is only the beginning.

So, based on the 2017 Annual Scientific Sessions, our patients with AF or those high-risk patients without can diagnose themselves with AF using their smartwatch and take their NOAC accordingly, and by doing so, prevent dementia. If they can’t take OAC, they can have LAA exclusion with simply antiplatelet therapy.

### Antiarrhythmic therapy post-ablation for AF

Abstracts on catheter ablation for AF once again were a large part of the 2017 Annual Scientific Sessions, but I do not think that I’m unique in my opinion that we are in a period of refinement of this procedure (specifically with respect to energy sources, lesion sets, targets and protocols) while we wait for the “next big thing.” Not to completely leave out catheter ablation, the results of the POWDER AF trial were presented at the late breaking clinical trials by Dr. Mattias Duytschaever.^12^ It is certainly true that most people seek the definition of ablation success that is reported in the literature—that is, when the patient is arrhythmia-free beyond the blanking period off antiarrhythmic drugs (AADs). This is arbitrary considering that a previously ineffective medication may become effective after a catheter ablation. The POWDER AF trial randomized patients undergoing their first contact force-guided ablation procedure who were free of arrhythmia during the three-month blanking period to either continuation or discontinuation of their previously ineffective AADs. At 12 months post ablation, a significantly higher percentage of patients remained arrhythmia-free as compared with those who stopped their AADs at three months (97.3% vs. 78.1%, p<0.001). Not surprisingly, patients had fewer unscheduled visits and improved symptoms. The authors conclude that combined therapy after a first ablation may result in improved outcomes.

While not surprising, the results of this study raise an important issue regarding clinical practice. We typically follow prior clinical trial design, which is designated primarily to demonstrate a benefit or superiority of a therapy. A clinical benefit such as that demonstrated in the POWDER AF trial should not be discounted. In addition, it will be interesting to see if there is a long-term benefit if AAD use is discontinued. This would be reminiscent of data originally presented at the Annual Sessions in which the AF-free period after epicardial fat pad botulinum toxin injection in patients undergoing open heart surgery long outlasted the autonomic effects.^13^

## Conclusions

AF has been a robust subject at many of the Heart Rhythm Sessions, and the 2017 Scientific Sessions were no exception. I hope these “highlights,” as subjective as they may be, spark a continuing interest in AF investigation, but more importantly, that they translate into improved care for our patients.

Rahul N. Doshi, MD, FHRS

rahuldoshimd@me.com

Associate Professor of Clinical Medicine

Director of Cardiac Electrophysiology

Keck School of Medicine

University of Southern California

Los Angeles, CA 90033
